# Deep learning for 3D cephalometric landmarking with heterogeneous multi-center CBCT dataset

**DOI:** 10.1371/journal.pone.0305947

**Published:** 2024-06-25

**Authors:** Jaakko Sahlsten, Jorma Järnstedt, Joel Jaskari, Hanna Naukkarinen, Phattaranant Mahasantipiya, Arnon Charuakkra, Krista Vasankari, Ari Hietanen, Osku Sundqvist, Antti Lehtinen, Kimmo Kaski

**Affiliations:** 1 Department of Computer Science, Aalto University School of Science, Espoo, Finland; 2 Department of Radiology, Tampere University Hospital, Wellbeing Services County of Pirkanmaa, Tampere, Finland; 3 Faculty of Medicine and Health Technology, University of Tampere, Tampere, Finland; 4 Planmeca Oy, Helsinki, Finland; 5 Department of Oral Diseases, Tampere University Hospital, Tampere, Finland; 6 Department of Oral Biology and Diagnostic Sciences, Faculty of Dentistry, Chiang Mai University, Chiang Mai, Thailand; 7 Division of Oral and Maxillofacial Radiology, Department of Oral Biology and Diagnostic Sciences, Faculty of Dentistry, Chiang Mai University, Chiang Mai, Thailand; 8 The Alan Turing Institute, British Library, London, United Kingdom; University of Catania: Universita degli Studi di Catania, ITALY

## Abstract

Cephalometric analysis is critically important and common procedure prior to orthodontic treatment and orthognathic surgery. Recently, deep learning approaches have been proposed for automatic 3D cephalometric analysis based on landmarking from CBCT scans. However, these approaches have relied on uniform datasets from a single center or imaging device but without considering patient ethnicity. In addition, previous works have considered a limited number of clinically relevant cephalometric landmarks and the approaches were computationally infeasible, both impairing integration into clinical workflow. Here our aim is to analyze the clinical applicability of a light-weight deep learning neural network for fast localization of 46 clinically significant cephalometric landmarks with multi-center, multi-ethnic, and multi-device data consisting of 309 CBCT scans from Finnish and Thai patients. The localization performance of our approach resulted in the mean distance of 1.99 ± 1.55 mm for the Finnish cohort and 1.96 ± 1.25 mm for the Thai cohort. This performance turned out to be clinically significant i.e., ≤ 2 mm with 61.7% and 64.3% of the landmarks with Finnish and Thai cohorts, respectively. Furthermore, the estimated landmarks were used to measure cephalometric characteristics successfully i.e., with ≤ 2 mm or ≤ 2° error, on 85.9% of the Finnish and 74.4% of the Thai cases. Between the two patient cohorts, 33 of the landmarks and all cephalometric characteristics had no statistically significant difference (p < 0.05) measured by the Mann-Whitney U test with Benjamini–Hochberg correction. Moreover, our method is found to be computationally light, i.e., providing the predictions with the mean duration of 0.77 s and 2.27 s with single machine GPU and CPU computing, respectively. Our findings advocate for the inclusion of this method into clinical settings based on its technical feasibility and robustness across varied clinical datasets.

## Introduction

Cephalometric analysis plays a critical role in the accurate treatment planning of malocclusions [1] as well as in guiding orthodontic treatment strategies and possible followup orthognathic surgeries [2, 3]. By quantifying the severity of skeletal discrepancies and identifying specific anatomical features that contribute to malocclusions, cephalometric analysis helps orthodontists and oral surgeons to tailor personalized treatment plans. This analysis provides linear and angular measurements of cephalometric landmarks and their relative positions, typically obtained from standard 2D cephalometric radiographs [4]. In cases where malocclusion or misalignment is the primary problem, orthodontic treatment alone may be sufficient without the need for surgical intervention [5, 6]. In young patients, it is important to identify the type of mandibular retrusion and to stimulate mandibular growth during puberty [7] in order to prevent the need for surgery later in life. Early intervention with functional orthodontic appliances has been shown to be successful in treating a cohort of patients with involvement of temporomandibular joint (TMJ) affected by juvenile idiopathic arthritis [8]. It also suggests that early intervention is recommended to prevent unfavorable facial development, including severe mandibular retrognathia, micrognathia, TMJ dysfunction, and severe periodontal loss [8]. In the case of oral and maxillofacial deformities of individuals who have reached skeletal maturity [9] and have severe skeletal discrepancies or structural abnormalities as well as functional problems with the masticatory system, orthognathic surgery is often performed to correct the function and appearance of the facial skeleton [10]. This complex surgery is always preceded by orthognathic surgical planning and is often accompanied by pre- and post-operative orthodontic treatment, all of which require cephalometric analysis. The decision to perform orthognathic surgery is often driven by a variety of clinical considerations [11]. Understanding the clinical indications and rationale for orthognathic surgery is essential to optimise patient outcomes and ensure comprehensive multidisciplinary care.

Recently, the use of 3D cephalometric analysis and 3D computer-assisted surgical (CAS) planning in orthognathic surgery has been increased, both providing an enhanced efficacy and accuracy in osteotomy reporting compared to the conventional 2D-based planning methods. CAS orthognathic planning uses the fusion of 3D CT or CBCT images and dental arch images in addition to the 2D cephalometric measurements. The dental arch images can be obtained from plaster cast or intraoral scans for direct digital optical impressions of the dental arch. However, such a fusion of multimodal information requires accurate and reproducible reference points to reduce surgical inaccuracies [12, 13]. In addition, the manual landmarking is laborious, time-consuming, and affected by clinically significant interobserver variability [14]. Furthermore, 3D cephalometry has been shown to improve treatment, diagnosis, and planning in orthognathic surgery patients with asymmetries using the extended McNamara analysis and Steiner’s Ricketts analysis [14, 15]. In patients with asymmetry, the advantage of 3D cephalometry over the traditional 2D method is that the left and right gonial angle sides of the CBCT image can be marked separately. Moreover, the manual marking of the landmarks is laborious, time-consuming, and affected by clinically significant interobserver variability [16].

Recently, multiple deep learning -based approaches have been introduced for automatic localization of cephalometric landmarks with clinically significant accuracy [17]. Among the deep learning -based cephalometric landmarking studies [18, 19], the early works focused on localizing the landmarks from 2D cephalograms [20–24] and the more recent works have also examined CT imaging for 3D cephalometric landmarking [16, 25–29]. The CBCT modality has also been gaining popularity for cephalometric landmark detection with deep learning [17, 30–36]. It has become widely used in recent years due to the lower radiation dose in comparison to CT [37].

The number of publications in the literature for machine learning based 3D cephalometric analysis using CT or CBCT modality is still quite limited and they mostly utilize small, non-clinical, or homogeneous datasets as indicated in Serafin et al. [38] and references therein. Specifically, some mainly focused on algorithmic development with homogeneous publicly available data, such as the Public Domain Database for Computational Anatomy [35, 36]. Others have used clinical data with heterogeneities such as jaw deformities but have not provided information of the CBCT scanners and their vendors, as well as limited to no information about the imaging parameters used [31–34, 39–41]. There has been one study with both a heterogeneous dataset and information about the scanner, but the data originated from a single device and from one institution [17]. In addition, previous works have had at most 61 CBCT scans for testing the algorithms [17]. As these studies utilize homogeneous and small datasets in their machine learning analysis, they may result in a biased estimation for the generalizability performance [42]. To demonstrate the generalizability of the deep learning -based automatic localization of cephalometric landmarks, it is of paramount importance to evaluate the method with large multi-vendor and multi-center datasets including different ethnic and geographical variations [43–45]. Furthermore, from the healthcare usability point of view, the prior work has either considered a limited number of clinically relevant landmarks [31, 35] or the deep learning approaches require considerable computation for inference [17, 31].

The aim of our study is to evaluate the 3D cephalometric landmarking performance of a deep learning system in a novel and clinically relevant setting. This is achieved by using so far the largest dataset consisting of multi-center, multi-provider, and multi-ethnic heterogeneous CBCT scans, which were annotated for numerous clinically important cephalometric landmarks including novel landmark groups. Furthermore, the proposed deep learning system can be integrated into clinical workflow due to low computing requirements, which further highlights the clinical relevance of our study.

## Materials and methods

In this section we describe the CBCT dataset, deep learning model, and evaluation metrics used in the study.

### Patient data

The CBCT imaging data was retrospectively collected from the Cranio and Dentomaxillofacial Radiological Department of the University Hospital of Tampere (TAUH), Finland and Department of Oral Radiology, Faculty of Dentistry, Chiang Mai University (CMU), Thailand. The datasets are not publicly available and restriction apply to their use. For the Finnish dataset the restrictions are imposed by the Wellbeing Services County of Pirkanmaa, Tampere University Hospital, Research services, P.O.Box 2000, 33521 Tampere, Finland (requests for the data access can be made to clinicaltrials@pirha.fi). For the Thai data the restrictions are imposed by the Chiang Mai University Research Ethic Committee, 239 Huay Kaew Road, Suthep Sub-district, Mueang District, Chiang Mai Province 50200 (requests for the data access can be made to cmurec.cmu@gmail.com). Both sets of data consisted of full facial scans from normal clinical workflows of orthognathic or facial surgery patients, including patients with normal anatomy as well as with anatomical deformities. In total, 309 CBCT scans have been collected with 199 from TAUH from two devices and 110 from CMU from one device, respectively. The distribution of scanning devices is described in Table 1.

**Table 1:** Distribution of scans used to develop and evaluate the method.

Cohort	Manufacturer	Scanner	Voxel spacing	Scans
Thai	NSTDA	DentiScan	0.25	6 (2%)
			0.4	104 (34%)
Finnish	Planmeca	Viso G7	0.15	2 (1%)
			0.2	102 (33%)
			0.3	48 (16%)
			0.45	2 (1%)
	Soredex	Scanora 3Dx	0.2	40 (13%)
			0.3	5 (2%)

**Fig 1:**
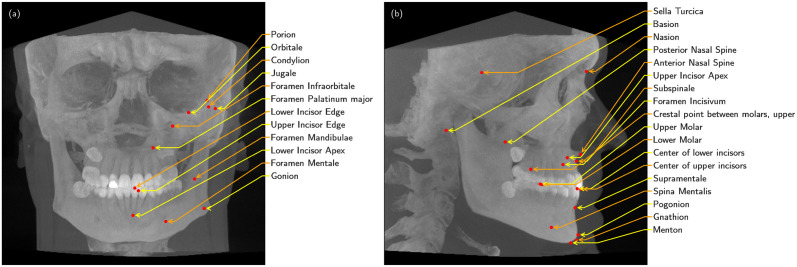
Visualization of the annotated landmarks overlaid on a maximum intensity projection of a CBCT. (a) Left side landmarks from the laterally symmetric pairs. (b) Midline landmarks.

The set of cephalometric landmarks was selected to enable the largest number of cephalometric analysis methods with a reasonable number of landmarks that cover the classic 2D cephalometric landmarks and marking points for dental arch image fusion. In addition, we emphasized selecting unambiguous and stable landmarks that do not change due to operations on the dentition, such as Foramen Infraorbitale, Palatinum Major, Incisivum, and Foramen Mentale. The dataset was annotated for 46 cephalometric landmarks. There are 16 bilaterally symmetric landmarks: Condylion (Co), Foramen Infraorbitale (FInf), Foramen Mandibulae (FMan), Foramen Mentale (FMen), Foramen Palatinum major (FPal), Gonion (Go), Jugale (J), Lower Incisor Apex (LIAL), Lower Incisor Edge (LIE), Lower Molar (LMol), Orbitale (Or), Crestal point between molars, upper left (PcmU), Porion (Po), Upper Incisor Apex (UIA), Upper Incisor Edge (UIE), and Upper Molar (UMol). In addition, there are 14 midline landmarks: Subspinale (A), Anterior Nasal Spine (ANS), Supramentale (B), Basion (Ba), Center of lower incisors (CLI), Center of upper incisors (CUI), Foramen Incisivum (FI), Gnathion (Gn), Menton (Me), Nasion (N), Posterior Nasal Spine (PNS), Pogonion (Pog), Sella Turcica (S), and Spina Mentalis (SM). The landmarks are visualized in Fig 1.

For the cephalometric analysis, we derived 16 cephalometric characteristic measures from the landmarks. Of these 12 were defined in the extended McNamara analysis [14] and four from traditional cephalometric analysis. The selected measures that can be grouped to three types. The first group includes five 3D point-to-point distance measures i.e., the left and right effective mandibular lengths, left and right effective midfacial length, and anterior facial height. The second group included six components of vector measures i.e., Nasion perpendicular to point A, Pogonion to Nasion perpendicular, right and left Upper Incisor point A verticals, as well as left and right Lower Incisor to point A pogonion line. The third group included five measurements of plane-to-plane angles i.e., Sella Turcica-Nasion-Subspinale (SNA) angle and Sella Turcica-Nasion-Supramentale (SNB) angle, and the Mandibular Plane angle which is measured between the mandibular plane (defined by Menton, and the Gonions) and Frankfort horizontal plane which is defined by the best orthogonal least squares fit between the visible Porions and Orbitales. In addition, the plane-to-plane angle between Frankfort horizontal and true horizontal planes are evaluated within the third group due to its importance in the cephalometric analysis.

The Finnish cohort was annotated by a specialist in dental and maxillofacial radiology, a resident in orthodontics with several years of experience in surgical planning using cephalometric landmarks and a resident in dental and maxillofacial radiology with several years of experience in 2D and 3D cephalometry using Romexis 4.6.2 software (Planmeca Oy, Helsinki, Finland). The Thai cohort was annotated by two specialists in dental and maxillofacial radiology with several years of experience in 2D cephalometry using OnDemand 3D software (Cybermed Co., Seoul, Korea).

In order to develop and evaluate the model, the dataset was split to training, validation, and test sets with 178 (58%), 28 (9%), and 103 (33%) scans with same number of patients, respectively. The training dataset included in total 8119 landmarks, validation dataset 1276 landmarks, and test dataset 4683 landmarks. The model parameters were optimized based on the training set, and the model hyperparameters, such as learning rate, were selected based on the validation set performance. Finally, our main results were calculated using the test set. The test set included 81 (79%) Finnish patient scans with 77 high resolution, one standard resolution, and 3 ultra-low dosage scans as well as 22 (21%) Thai patient all with standard resolution dosage. Within the test set, the Finnish cohort scans included 72 Planmeca Viso G7 scans and 9 Soredex Scanora 3Dx scans, and all the Thai cohort scans were taken using the National Science and Technology Development Agency (NSTDA) DentiScan.

This study is based on a retrospective and registration dataset and as such does not involve experiments on humans and/or the use of human tissue samples and no patients were imaged for this study. A registration and retrospective study does not need ethical permission or informed consent from subjects according to the law of Finland (Medical Research Act (488/1999) and Act on Secondary Use of Health and Social Data (552/2019)) and according to European General Data Protection Regulation (GDPR) rules 216/679. The use of the Finnish imaging data was accepted by the Tampere University Hospital Research Director, Finland October 1, 2019 (vote number R20558). Certificate of Ethical Clearance for the Thai imaging data was given by the Human Experimentation Committee, Faculty of Dentistry, Chiang Mai University, Thailand (vote number 33/2021) July 5, 2021. According to the Thailand legislation informed consent was not needed. The data was accessed on July 5, 2021 and the authors did not have access to identifying information.

### Deep learning model

**Fig 2:**
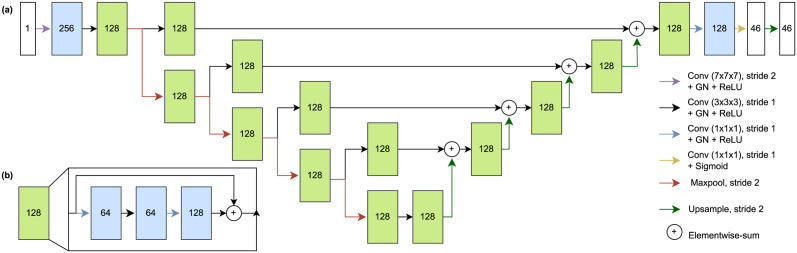
The architecture of the deep learning model a. A U-net style deep learning system architecture with a contracting pathway, an expanding pathway, and connecting pathways between. b. Single residual block consists of three convolutions, each followed by group normalization (GN) and ReLU nonlinearity with elementwise summation.

The deep learning model of this study is based on the stacked hourglass network [46]. Our experiments utilize the single stacked variant with the first convolution block having 256 features, kernel size of 7 and stride of 2, with latter residual convolution blocks having 64, 64, and 128 features with kernel size of 1, 1, and 3, respectively. All convolutions, except the last one, are followed by a group normalization layer with 8 groups and ReLU non-linearity. The last convolution has no normalization layer and uses sigmoid type non-linearity. Feature maps are downsampled with maxpooling using stride of 2 in the contracting pathway and upsampled using trilinear sampling with stride of 2 in the expanding pathway. The model architecture is depicted in Fig 2 uses a single input channel and produces 46 output segmentation maps, one for each landmark. The model is trained using full sized CBCT images that are resampled into 1.6 mm isotropic voxel spacing with the target of Gaussian sphere heatpoint segmentation map being centered on the landmark coordinates with *σ* = 1.5. Final coordinates are calculated using the center-of-mass operation of SciPy (1.7.3) library [47]. The model was trained using binary cross-entropy loss until convergence with model selection using the checkpoint of model parameters with lowest average Euclidean distance of all the landmarks on the validation set. Parameter updates were calculated using AdamW optimizer (*γ* = 0.001, λ = 0.01). During the training process, the training data was augmented using the random crops (0–75% each axis), local elastic deformation, random rotation (−15°, 15°), random translation (−10%, 10%), and random contrast and brightness changes.

### Evaluation metrics

As the main evaluation metric for the localization performance, we use the following Euclidean distance:

$$\begin{array}{c} d\left(t,p\right)=\sqrt{{\left(t_{x}-p_{x}\right)}^{2}+{\left(t_{y}-p_{y}\right)}^{2}+{\left(t_{z}-p_{z}\right)}^{2}}, \end{array}$$

where *t* and *p* are the target and predicted coordinates, respectively. The localization performance is considered clinically usable when the Euclidean distance is ≤ 2 mm [48]. The rate of clinically usable cases is defined as the successful detection rate (SDR) measure. The statistical significance tests between the cohort and device groups are based on the Mann-Whitney U test with Benjamini–Hochberg procedure to correct for multiple testing, in which p < 0.05 is considered significant. In addition, prior to main evaluation a qualitative analysis of the largest errors in the test set was made by a radiologist visually inspecting the landmarks and categorizing each one of them as subjective difference or as an objective annotation error which were included or discarded from the final analysis, respectively.

For the cephalometric characteristic measures, we evaluate either the Euclidean distance between two landmarks projected into a 2D plane or in 3D. In addition, the angle measures are calculated between two landmarks or planes. A cephalometric characteristic measure is considered to be successful with for the distance being ≤ 2 mm or angle being ≤ 2°.

The performance of model inference was evaluated as the duration of producing the 46 landmark coordinates for a single scan. In this analysis, we evaluate the inference performance for all the 309 scans. The inference performance is evaluated in two experiments, i.e., one with a CPU only setup and another using both CPU and GPU. All the experiments are performed with a single machine using a GTX 1080 Ti GPU and Intel i7–6850K CPU.

## Results

In the qualitative analysis, all landmarks with the prediction to target differences above 10 mm (N = 40) were inspected by senior radiologists. It turned out that 35 of these differences were caused by an objective annotation error, while 5 of the differences were caused by the DLS error. The annotation errors included landmark pairs that were in incorrect order (N = 6), dental landmarks on incorrect teeth (N = 10), annotator misinterpretations (N = 10), and incorrectly localized markings (N = 9). In the following analysis, the incorrectly ordered landmarks (N = 6) were corrected and included while the landmarks with the other annotator errors (N = 29) were not included. The results prior to the filtering based on the qualitative analysis are reported in the Figs B and C in S1 Appendix.

**Fig 3:**
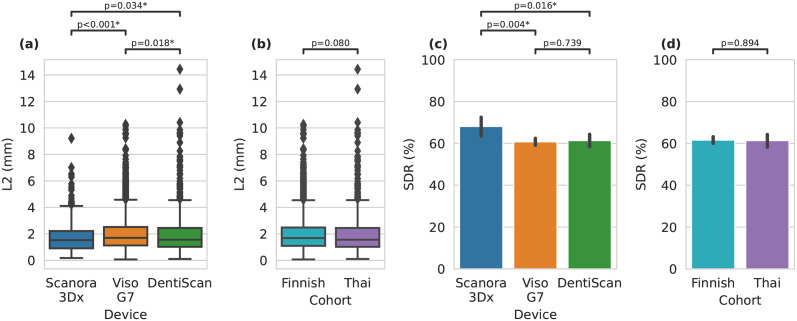
Boxplots of landmarking results evaluated with Euclidean distance when grouping by (a) device and (b) cohort. Barplots landmarking success detection rate (SDR) when grouping by (c) device and (d) cohort. Statistical significance was determined using the Mann-Whitney U test with Benjamini–Hochberg correction procedure.

In terms of device specific comparisons, the method performed with statistically significant difference for both the distance and SDR on all devices except for Viso G7 and DentiScan in the case of SDR. Specifically, the Scanora 3Dx scans resulted in the mean distance of 1.77 ± 1.20 mm and SDR of 68.1%, the Viso G7 the mean distance of 1.99 ± 1.26 mm with 60.8% SDR, and the DentiScan the mean distance of 1.99 ± 1.55 mm and SDR of 61.3%. When comparing the two cohorts, no statistically significant differences were found for both evaluating the distance and SDR. Specifically, the Finnish scans had the mean distance of 1.96 ± 1.25 mm and SDR of 61.7%, and the Thai scans had the mean distance of 1.99 ± 1.55 mm and SDR of 64.3%. The full comparisons with the device or cohort grouping are depicted in Fig 3. Additional cohort comparison results when grouped by landmark group type, radiation dosage, and location on bony surface are reported in Fig A in S1 Appendix. Moreover, the robustness of landmarking performance when evaluated on partial volumes is reported in Table A in S1 Appendix.

**Fig 4:**
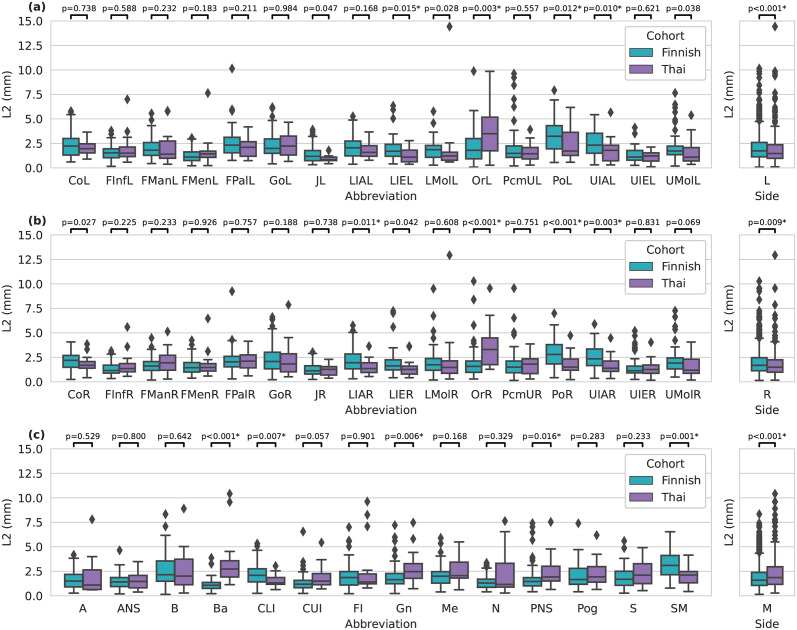
Boxplot of landmarking results with each row having (a) the left, (b) right and (c) midline landmarks shown individually and as a group. Statistical significance was determined using the Mann-Whitney U test with Benjamini–Hochberg correction procedure. ^*)^statistically significant difference (p < 0.05).

In terms of landmark specific results, for the Finnish cohort the mean distances turned out to be 2.04 ± 1.31 mm, 1.95 ± 1.22 mm, 1.90 ± 1.22 mm for the left, right and midline landmarks, respectively. For the Thai cohort the left, right and midline landmarks had the mean values of 1.91 ± 1.61 mm, 1.83 ± 1.35 mm, 2.27 ± 1.65 mm, respectively. When grouped by the left and middle sides the landmarks had statistically significant difference between the two cohorts with (p < 0.05). Overall, there was 13 statistically significant differences (p < 0.05) between the two cohorts. The smallest and the largest median error for the Finnish cohort turned out to be for the landmarks Ba and PoL having the median (IQR) values of 1.07 (0.60) mm and 3.23 (2.37) mm, respectively. For the Thai cohort the smallest median error was 0.84 (0.35) mm with the JL and the largest error was 3.49 (3.43) mm for the OrL. The smallest median difference between these two cohorts was for the Right Foramen Mentale with median (IQR) values of 1.45 (0.97) mm and 1.46 (0.75) mm, for the Finnish and Thai cohort, respectively. The largest median difference between the two cohorts turned out to be for the OrR landmarks having the median (IQR) values of 1.58 (1.16) mm and 3.30 (2.70) mm with the with the Finnish cohort and Thai cohort, respectively. The full results for all the landmarks are reported in Fig 4 and Table 2.

**Table 2:** Comparison of the mean and standard deviation of distances and successful detection rate for the landmarks between the cohorts.

Group	Landmark	Distance (mm)			SDR (%)	
		Finnish cohort	Thai cohort	p-value	Finnish cohort	Thai cohort
Dental	(CLI) Center of lower incisors	2.1 ± 1.1	1.5 ± 0.6	0.007*	44.4	68.2
	(CUI) Center of upper incisors	1.3 ± 0.9	1.8 ± 1.2	0.057	86.4	68.2
	(LIAL) Lower Incisor Apex Left	2.1 ± 1.1	1.7 ± 0.8	0.168	48.1	59.1
	(LIAR) Lower Incisor Apex Right	2.2 ± 1.2	1.5 ± 0.8	0.011*	51.9	72.7
	(LIEL) Lower Incisor Edge Left	1.9 ± 1.1	1.3 ± 0.8	0.015*	66.7	63.6
	(LIER) Lower Incisor Edge Right	1.8 ± 1.2	1.4 ± 0.9	0.042	67.9	81.8
	(LMolL) Lower Molar Left	1.8 ± 0.9	1.9 ± 2.9	0.028	60.5	81.8
	(LMolR) Lower Molar Right	1.9 ± 1.4	2.2 ± 2.7	0.608	58.0	54.5
	(PcmUL) Crestal point between molars, upper left	2.1 ± 1.9	1.6 ± 0.9	0.557	65.4	68.2
	(PcmUR) Crestal point between molars, upper right	1.8 ± 1.4	1.7 ± 1.0	0.751	74.1	63.6
	(UIAL) Upper Incisor Apex Left	2.5 ± 1.2	1.7 ± 1.2	0.010*	40.7	68.2
	(UIAR) Upper Incisor Apex Right	2.5 ± 1.2	1.7 ± 1.0	0.003*	42.0	63.6
Facial surgery	(FI) Foramen Incisivum	2.0 ± 1.3	2.4 ± 2.5	0.901	61.7	72.7
	(FInfL) Foramen Infraorbitale Left	1.6 ± 0.8	1.9 ± 1.5	0.588	76.5	63.6
	(FInfR) Foramen Infraorbitale Right	1.3 ± 0.7	1.7 ± 1.1	0.225	84.0	72.7
	(FManL) Foramen Mandibulae Left	2.0 ± 1.1	1.9 ± 1.5	0.232	60.5	68.2
	(FManR) Foramen Mandibulae Right	1.7 ± 0.8	2.0 ± 1.2	0.233	70.4	50.0
	(FMenL) Foramen Mentale Left	1.2 ± 0.6	1.7 ± 1.5	0.183	87.7	81.8
	(FMenR) Foramen Mentale Right	1.6 ± 0.8	1.7 ± 1.2	0.926	76.5	77.3
	(FPalL) Foramen Palatinum major Left	2.5 ± 1.4	2.1 ± 1.1	0.211	37.0	50.0
	(FPalR) Foramen Palatinum major Right	2.2 ± 1.2	2.2 ± 0.9	0.757	48.1	36.4
Skeletal	(A) Subspinale	1.7 ± 1.0	1.8 ± 1.7	0.529	65.4	68.2
	(ANS) Anterior Nasal Spine	1.5 ± 0.8	1.6 ± 0.9	0.800	76.5	72.7
	(B) Supramentale	2.6 ± 1.6	2.6 ± 2.0	0.642	44.4	45.5
	(Ba) Basion	1.1 ± 0.6	3.3 ± 2.4	<0.001*	96.3	31.8
	(CoL) Condylion Left	2.3 ± 1.2	2.1 ± 0.9	0.738	48.1	50.0
	(CoR) Condylion Right	2.2 ± 0.9	1.8 ± 0.8	0.027	40.7	63.6
	(Gn) Gnathion	1.9 ± 1.2	2.8 ± 1.6	0.006*	65.4	31.8
	(GoL) Left Gonion	2.4 ± 1.3	2.4 ± 1.3	0.984	50.6	45.5
	(GoR) Right Gonion	2.5 ± 1.5	2.2 ± 1.7	0.188	44.4	68.2
	(JL) Jugale Left	1.3 ± 0.8	0.9 ± 0.3	0.047	84.0	86.4
	(JR) Jugale Right	1.3 ± 0.6	1.2 ± 0.6	0.738	87.7	81.8
	(Me) Menton	2.1 ± 1.1	2.6 ± 1.3	0.168	50.6	50.0
	(N) Nasion	1.4 ± 0.7	2.3 ± 2.1	0.329	85.2	59.1
	(OrL) Left Orbitale	2.1 ± 1.5	4.4 ± 3.1	0.003*	55.6	22.7
	(OrR) Right Orbitale	1.9 ± 1.5	3.7 ± 2.3	<0.001*	70.4	22.7
	(PNS) Posterior Nasal Spine	1.8 ± 1.3	2.4 ± 1.6	0.016*	77.8	54.5
	(PoL) Left Porion	3.3 ± 1.6	2.4 ± 1.6	0.012*	25.9	59.1
	(PoR) Right Porion	2.8 ± 1.3	1.8 ± 1.0	<0.001*	33.3	68.2
	(Pog) Pogonion	2.1 ± 1.2	2.3 ± 1.3	0.283	59.3	54.5
	(S) Sella Turcica	1.8 ± 1.0	2.3 ± 1.4	0.233	58.0	45.5
	(SM) Spina Mentalis	3.1 ± 1.4	2.0 ± 0.9	0.001*	22.2	45.5
	(UIEL) Upper Incisor Edge Left	1.3 ± 0.8	1.1 ± 0.5	0.621	86.4	95.5
	(UIER) Upper Incisor Edge Right	1.4 ± 1.0	1.4 ± 0.8	0.831	80.2	86.4
	(UMolL) Upper Molar Left	2.1 ± 1.4	1.6 ± 1.3	0.038	63.0	63.6
	(UMolR) Upper Molar Right	2.1 ± 1.2	1.6 ± 1.0	0.069	53.1	63.6

^*)^ Statistically significant differences (p < 0.05) evaluated with the Mann-Whitney U test with the Benjamini–Hochberg correction procedure.

**Fig 5:**
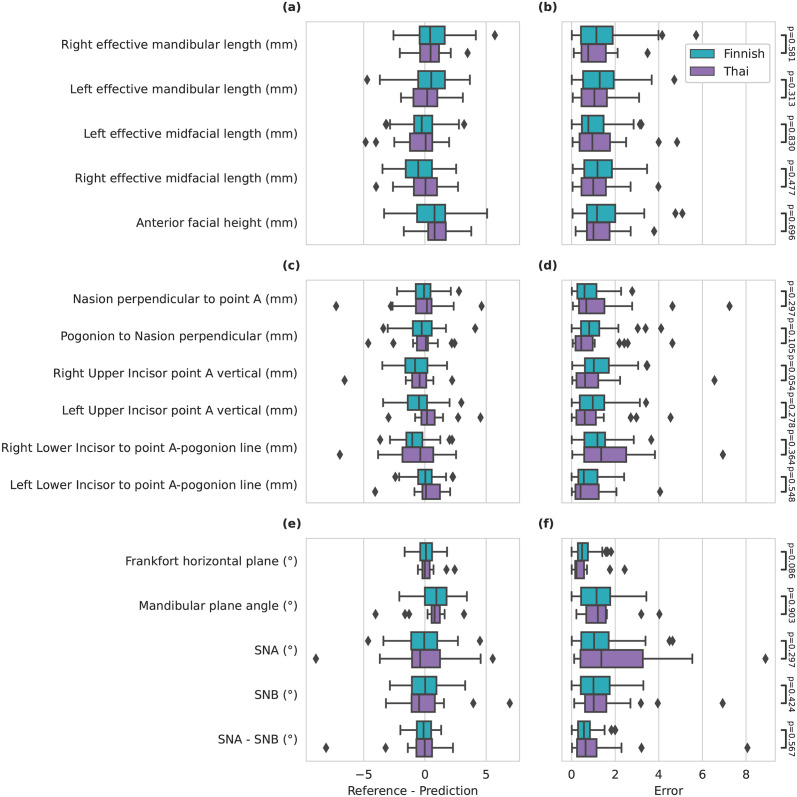
Boxplot of performance on cephalometric characteristic measures with 3D point-to-point measures with (a) reference to prediction distance and (b) error, components of vector with (c) reference to prediction distance and (d) error, and plane-to-plane angle with (e) reference to prediction distance and (f) error. Cohort difference between each error measures are compared with the Mann-Whitney U test with Benjamini–Hochberg correction procedure.

For the cephalometric characteristic measures the SDR on all 15 measures turned out to be 84.0% for the Finnish cohort and the mean values for 3D point-to-point, components of a vector, and plane-to-plane angle measures was 1.3 ± 1.0 mm, 1.2 ± 0.9 mm, and 1.0 ± 0.8°, respectively. For the Thai cohort the average SDR was 71.3% and the mean values for 3D point-to-point, components of a vector, and plane-to-plane angle measures was 1.2 ± 1.0 mm, 1.6 ± 1.7 mm, and 1.4 ± 1.6°, respectively. The lowest performing measure was Pogonion to Nasion perpendicular with the Finnish cohort having on average error of 1.8 ± 1.1 mm and 70.4% SDR, and the Thai cohort having on 2.7 ± 2.0 mm error and 40.9% SDR. The best performing measure was SNA—SNB with 0.6 ± 0.4° error and 97.5% SDR on the Finnish cohort and Frankfort horizontal plane on Thai cohort with 0.8 ± 0.9 mm and 72.7% SDR on the Thai cohort. The full results are reported in the Fig 5 and Table 3.

**Table 3:** Mean and standard deviation of distances and successful detection rate (SDR) from reference to prediction (Ref.—Pred.) for the cephalometric characteristics. SDR is defined as ≤2 mm or ≤2° of the absolute error.

Type	Characteristic	Finnish cohort		Thai cohort	
		Ref.—Pred. (mm)	SDR (%)	Ref.—Pred. (mm)	SDR (%)
3D point-to-point (mm)	Anterior facial height	0.6 ± 1.7	74.1	0.9 ± 1.4	77.3
	Left effective mandibular length	0.5 ± 1.6	80.2	0.2 ± 1.3	90.9
	Left effective midfacial length	-0.2 ± 1.3	86.4	-0.4 ± 1.8	81.8
	Right effective mandibular length	0.7 ± 1.6	76.5	0.4 ± 1.3	81.8
	Right effective midfacial length	-0.5 ± 1.4	82.7	-0.2 ± 1.6	77.3
Components of a vector (mm)	Nasion perpendicular to point A	1.3 ± 0.7	86.4	2.2 ± 2.0	59.1
	Pogonion to Nasion perpendicular	1.8 ± 1.1	70.4	2.7 ± 2.0	40.9
	Right Upper Incisor point A vertical	-0.7 ± 1.3	81.5	-0.6 ± 1.7	72.7
	Left Upper Incisor point A vertical	-0.5 ± 1.2	86.4	0.4 ± 1.5	68.2
	Right Lower Incisor to point A-pogonion line	-0.8 ± 1.2	81.5	-0.8 ± 2.3	59.1
	Left Lower Incisor to point A-pogonion line	-0.0 ± 1.0	95.1	0.2 ± 1.3	81.8
Plane-to-plane angle (°)	Frankfort horizontal plane	0.9 ± 0.4	98.8	0.8 ± 0.9	72.7
	Mandibular plane angle	0.8 ± 1.2	82.7	0.5 ± 1.4	63.6
	SNA	-0.2 ± 1.6	81.5	-0.4 ± 3.1	63.6
	SNB	-0.0 ± 1.4	81.5	-0.0 ± 2.2	72.7
	SNA—SNB	-0.1 ± 0.8	97.5	-0.5 ± 2.1	77.3

The DLS pipeline was evaluated for inference performance when running GPU and CPU-only setups for all the 309 scans included in the study. The mean and standard deviation of inference duration was 0.77 ± 0.07s for the GPU and 2.27 ± 0.22s for the CPU only setup. Inference duration for all scans ranged from 0.29 to 0.79 s with the GPU setup and from 0.83 to 2.54 s with the CPU only setup.

## Discussion

In this study, we have developed a fast deep learning method for automatic 3D cephalometric analysis using multi-ethnic, multi-center, and multi-vendor CBCT scans to evaluate the robustness of our method with a large dataset including two heterogeneous cohorts. The method turned out to localize the landmarks with clinically sufficient precision and provide accurate cephalometric characteristic information in the majority of the cases for both cohorts. Furthermore, there was no statistically significant difference with 72% of the landmarks and 100% of the cephalometric characteristics between the two cohorts, demonstrating the robustness of the method. Our single-stage approach for the deep learning model provided computationally light automatic landmarking. Moreover, the ambiguity in the localization of landmarks between graders such as seen with the left and right Orbitales in our dataset highlights the need for standardization of the 3D cephalometric landmarking and the use of multiple graders or consensus.

Clinical usability of automatic 3D cephalometric analysis is dependent on the selection of landmarks, which lacks a standard as is evident in the previous works [17, 36]. In our analysis, we have evaluated the method for localizing three types of clinically relevant cephalometric landmarks. In the first skeletal group, the landmarks or cephalometric points are also used in conventional 2D cephalometric analysis. In the second dental group, the landmarks are used to determine tooth axes and angles in 2D and 3D cephalometric analysis, and some are also used for tooth segmentation and bite plane determination.

Accurate 3D cephalometric analysis enables standardization of protocols for treatment planning for orthodontics and orthognathic surgery. This ensures consistency [49], optimizes resources, and minimizes misdiagnosis risks that leads to better treatment outcomes [50] while misdiagnosis can result in suboptimal strategies such as relying on dental compensation instead of orthognathic surgery [2]. This is especially important for patients with periodontitis as it obscures malocclusions and results in inaccurate diagnoses leading to ineffective treatments [2], while malocclusions can exacerbate periodontitis [51].

The use of 3D cephalometric analysis and 3D computer-assisted surgical planning in orthognathic surgery has recently been increasing, providing greater efficiency and accuracy in osteotomy reporting compared to the conventional 2D-based planning methods. The dental arch images for CAS can be obtained from plaster casts or intraoral scans for direct digital optical impressions of the dental arch. However, such a fusion of multimodal information requires accurate and reproducible reference points to reduce surgical inaccuracies [12, 13]. From the dental group the Lower Incisor Edge, Lower Molar, Crestal point between molars, upper left, Upper Incisor Edge, Upper Molar, Center of lower incisors, and Center of upper incisors landmarks can be used to fuse surface models with the CBCT data. The third facial surgical group includes i.e., the Foramen Infraorbitale, Foramen Mandibulae, Foramen Mentale, Foramen Palatinum Major, and Foramen Incisivum are independent of the dentition. They reflect the shape of the face and the relationship between the jaws and can therefore be used to analyze the three-dimensional structure of the face. This analysis is essential for automated presetting of surgical planes in orthognathic surgery and for planning various other types of facial surgery, such as complex trauma or oral cancer reconstructive surgery. In the follow-up studies, these points can also be used to register slice sets for comparison and to identify anatomical landmarks in the development of new automated DLS models in the future.

In the initial qualitative analysis, the method turned out to have 40 errors that were larger than 10 mm caused by incorrect annotations or the DLS. However, in the test set of 4712 landmarks, there were only 35 errors (0.7%) and 5 (0.0%) for the annotators and the DLS, respectively. Moreover, the large errors for the DLS were on a Lower Molar point of a patient with missing lower incisors and premolars. The DLS performance including the annotation errors are reported in Figs B and C in S1 Appendix. For the landmarking performance, the method performed similarly to previous work with average 1.96 mm error for all valid landmarks with most of the landmarks being localized within clinically relevant accuracy i.e., ≤ 2 mm. The method was able to learn accurate landmarking for both cohorts as non-significant differences were found between them. This leads to the conclusion that the method can learn to handle heterogeneities of different cohorts even for patients of different ethnicity and being imaged with a different device. For the cephalometric measurements, we used the extended McNamara analysis, which has been shown to improve treatment, diagnosis and planning in orthognathic surgery patients with asymmetries [14]. The DLS turned out to have better success rate, i.e., ≤ 2 mm or ≤ 2°, on average with cephalometric measures to the landmarking. This shows that the measures are more robust to the landmarking errors. However, this is dependent on the selection of landmarks and the cephalometric analyses. Similarly to landmarking, the cephalometric measures showed no statistical differences between the two cohorts, showing the method’s ability to handle the two heterogeneities.

In our study some of the landmarks turned out to be challenging to annotate such as the Orbitale and Basion from the first group, and from the second group the Lower Molar, Upper Molar, Crestal point between molars from the second group. No considerable annotation errors were found in the third group. The Orbitale is the lowest point on the inferior border of the orbit, located on a curved surface, and therefore difficult to locate in 3D analysis using CBCT images, a similar finding as in a previous study [52]. In our study, one of the annotators placed the Orbitale at the bottom of the bony base of the orbit instead of the lowest point of the lower margin. A similar finding was seen in the second group of dental points, where annotators made errors by placing the Molar or Crestal points on the wrong molars. The findings highlight the importance of correct annotation in AI development and the potential for annotator bias. The limited number of DLS landmark position errors and the robustness of the cephalometric measurements indicate that the DLS can be successfully used in semi-automatic 3D cephalometric analysis. Prior to clinical use, an analysis with temporal and more multi-ethnic, multi-vendor CBCT data is required to evaluate the generalisability and repeatability of the method. It is also important to develop automated analysis for the fusion of dental arch images. This will enable the development of an efficient tool for computer-assisted surgical planning for orthognathic and facial surgery, which would facilitate the workflow for complex surgeries.

There are some limitations in our study. Although the dataset was annotated by multiple annotators to reduce bias, each scan was annotated only once without verification. This can lead to some errors some of which were found during the qualitative analysis. In addition, although our analysis utilized a multi-ethnic, multi-center, and multi-device dataset, the analysis was limited to the aggregate instead of examining each individually. Due to the limited samples, we did not include generalizability analysis between these datasets.

For clinical use it is necessary to repeat the analysis with temporal and more multi-ethnic, multi-vendor CBCT data to analyze the generalisability and repeatability of automated landmark detection models. It is also important to develop automated analysis for the fusion of dental arch images. This will enable the development of an efficient tool for computer-assisted surgical planning for orthognathic surgery, which would facilitate the workflow for complex surgeries.

## Conclusion

Accurate 3D cephalometric landmarking can improve treatment outcomes and patient quality of life through standardization of care, reduced misdiagnosis, and suitable treatment planning. Here we proposed a fast single-stage deep learning method for 3D cephalometric landmarking using multi-ethnic, multi-provider, and multi-center CBCT dataset for 46 clinically relevant landmarks. The method provided clinically sufficient accuracy in most of the predicted cases in terms of landmarking and subsequent cephalometric analysis with slight difference between the cohorts. The combination of the clinically significant accuracy and fast inference of the method enables follow-up studies in a practical setting.

## Supporting information

S1 Appendix Supplementary methods and results. (PDF)

## References

[1] Reyneke JP, Ferretti C. Diagnosis and planning in orthognathic surgery. Oral and maxillofacial surgery for the clinician. 2021; p. 1437–1462. doi: 10.1007/978-981-15-1346-6_66

[2] Dot G, Rafflenbeul F, Arbotto M, Gajny L, Rouch P, Schouman T. Accuracy and reliability of automatic three-dimensional cephalometric landmarking. International Journal of Oral and Maxillofacial Surgery. 2020;49(10):1367–1378. doi: 10.1016/j.ijom.2020.02.015 32169306

[3] Alsubai S. A critical review on the 3D cephalometric analysis using machine learning. Computers. 2022;11(11):154. doi: 10.3390/computers11110154

[4] Kusnoto B. Two-dimensional cephalometry and computerized orthognathic surgical treatment planning. Clinics in plastic surgery. 2007;34(3):417–426. doi: 10.1016/j.cps.2007.04.005 17692701

[5] Proffit WR, Phillips C, Douvartzidis N. A comparison of outcomes of orthodontic and surgical-orthodontic treatment of Class II malocclusion in adults. American Journal of Orthodontics and Dentofacial Orthopedics. 1992;101(6):556–565. doi: 10.1016/0889-5406(92)70131-S 1598896

[6] McNeill RW, West RA. Severe mandibular retrognathism: orthodontic versus surgical orthodontic treatment. American Journal of Orthodontics. 1977;72(2):176–182. doi: 10.1016/0002-9416(77)90058-6 268148

[7] Perillo L, Padricelli G, Isola G, Femiano F, Chiodini P, Mataresei G. Class II malocclusion division 1: a new classification method by cephalometric analysis. European journal of paediatric dentistry. 2012;13(3):192. 22971255

[8] Isola G, Ramaglia L, Cordasco G, Lucchese A, Fiorillo L, Matarese G. The effect of a functional appliance in the management of temporomandibular joint disorders in patients with juvenile idiopathic arthritis. Minerva stomatologica. 2016;66(1):1–8. 27716739 10.23736/S0926-4970.16.03995-3

[9] Weaver N, Glover K, Major P, Varnhagen C, Grace M. Age limitation on provision of orthopedic therapy and orthognathic surgery. American journal of orthodontics and dentofacial orthopedics. 1998;113(2):156–164. doi: 10.1016/S0889-5406(98)70287-2 9484206

[10] Naran S, Steinbacher DM, Taylor JA. Current concepts in orthognathic surgery. Plastic and reconstructive surgery. 2018;141(6):925e–936e. doi: 10.1097/PRS.0000000000004438 29794714

[11] Sabri R. Orthodontic objectives in orthognathic surgery: state of the art today. World journal of orthodontics. 2006;7(2). 16779977

[12] Haas O Jr, Becker O, De Oliveira R. Computer-aided planning in orthognathic surgery—systematic review. International journal of oral and maxillofacial surgery. 2015;44(3):329–342. doi: 10.1016/j.ijom.2014.10.025 25432508

[13] Swennen G, Mommaerts M, Abeloos J, De Clercq C, Lamoral P, Neyt N, et al. A cone-beam CT based technique to augment the 3D virtual skull model with a detailed dental surface. International journal of oral and maxillofacial surgery. 2009;38(1):48–57. doi: 10.1016/j.ijom.2008.11.006 19118978

[14] Dos Santos RMG, De Martino JM, Neto FH, Passeri LA. Cone-beam computed tomography-based three-dimensional McNamara cephalometric analysis. Journal of Craniofacial Surgery. 2018;29(4):895–899. doi: 10.1097/SCS.0000000000004248 29381618

[15] Gateno J, Xia JJ, Teichgraeber JF. New 3-dimensional cephalometric analysis for orthognathic surgery. Journal of oral and maxillofacial surgery. 2011;69(3):606–622. doi: 10.1016/j.joms.2010.09.010 21257250 PMC3059215

[16] Dot G, Schouman T, Chang S, Rafflenbeul F, Kerbrat A, Rouch P, et al. Automatic 3-dimensional cephalometric landmarking via deep learning. Journal of dental research. 2022;101(11):1380–1387. doi: 10.1177/00220345221112333 35982646

[17] Deng HH, Liu Q, Chen A, Kuang T, Yuan P, Gateno J, et al. Clinical feasibility of deep learning-based automatic head CBCT image segmentation and landmark detection in computer-aided surgical simulation for orthognathic surgery. International Journal of Oral and Maxillofacial Surgery. 2022. doi: 10.1016/j.ijom.2022.10.010 36372697 PMC10169531

[18] Juneja M, Garg P, Kaur R, Manocha P, Prateek, Batra S, et al. A review on cephalometric landmark detection techniques. Biomedical Signal Processing and Control. 2021;66:102486. doi: 10.1016/j.bspc.2021.102486

[19] Schwendicke F, Chaurasia A, Arsiwala L, Lee JH, Elhennawy K, Jost-Brinkmann PG, et al. Deep learning for cephalometric landmark detection: systematic review and meta-analysis. Clinical Oral Investigations. 2021;25(7):4299–4309. doi: 10.1007/s00784-021-03990-w 34046742 PMC8310492

[20] Lee H, Park M, Kim J. Cephalometric landmark detection in dental x-ray images using convolutional neural networks. In: Medical Imaging 2017: Computer-Aided Diagnosis. vol. 10134. International Society for Optics and Photonics. SPIE; 2017. p. 101341W. Available from: 10.1117/12.2255870.

[21] Chen R, Ma Y, Chen N, Lee D, Wang W. Cephalometric Landmark Detection by Attentive Feature Pyramid Fusion and Regression-Voting. In: Medical Image Computing and Computer Assisted Intervention—MICCAI 2019. Cham: Springer International Publishing; 2019. p. 873–881.

[22] Park JH, Hwang HW, Moon JH, Yu Y, Kim H, Her SB, et al. Automated identification of cephalometric landmarks: Part 1—Comparisons between the latest deep-learning methods YOLOV3 and SSD. The Angle Orthodontist. 2019;89(6):903–909. doi: 10.2319/022019-127.1 31282738 PMC8109157

[23] Song Y, Qiao X, Iwamoto Y, Chen Yw. Automatic Cephalometric Landmark Detection on X-ray Images Using a Deep-Learning Method. Applied Sciences. 2020;10(7). doi: 10.3390/app10072547

[24] Oh K, Oh IS, Le VNT, Lee DW. Deep Anatomical Context Feature Learning for Cephalometric Landmark Detection. IEEE Journal of Biomedical and Health Informatics. 2021;25(3):806–817. doi: 10.1109/JBHI.2020.3002582 32750939

[25] Lee SM, Kim HP, Jeon K, Lee SH, Seo JK. Automatic 3D cephalometric annotation system using shadowed 2D image-based machine learning. Physics in Medicine & Biology. 2019;64(5):055002. doi: 10.1088/1361-6560/ab00c9 30669128

[26] Ma Q, Kobayashi E, Fan B, Nakagawa K, Sakuma I, Masamune K, et al. Automatic 3D landmarking model using patch-based deep neural networks for CT image of oral and maxillofacial surgery. The International Journal of Medical Robotics and Computer Assisted Surgery. 2020;16(3):e2093. doi: 10.1002/rcs.2093 32065718

[27] Yun HS, Jang TJ, Lee SM, Lee SH, Seo JK. Learning-based local-to-global landmark annotation for automatic 3D cephalometry. Physics in Medicine & Biology. 2020;65(8):085018. doi: 10.1088/1361-6560/ab7a71 32101805

[28] Kang SH, Jeon K, Kang SH, Lee SH. 3D cephalometric landmark detection by multiple stage deep reinforcement learning. Scientific Reports. 2021;11(1):17509. doi: 10.1038/s41598-021-97116-7 34471202 PMC8410904

[29] Yun HS, Hyun CM, Baek SH, Lee SH, Seo JK. A semi-supervised learning approach for automated 3D cephalometric landmark identification using computed tomography. PLOS ONE. 2022;17(9):1–18. doi: 10.1371/journal.pone.0275114 36170279 PMC9518928

[30] Zhang J, Liu M, Wang L, Chen S, Yuan P, Li J, et al. Joint Craniomaxillofacial Bone Segmentation and Landmark Digitization by Context-Guided Fully Convolutional Networks. In: Descoteaux M, Maier-Hein L, Franz A, Jannin P, Collins DL, Duchesne S, editors. Medical Image Computing and Computer-Assisted Intervention—MICCAI 2017. Cham: Springer International Publishing; 2017. p. 720–728. 10.1007/978-3-319-66185-8_81 PMC5786437 29376150

[31] Zhang J, Liu M, Wang L, Chen S, Yuan P, Li J, et al. Context-guided fully convolutional networks for joint craniomaxillofacial bone segmentation and landmark digitization. Medical Image Analysis. 2020;60:101621. doi: 10.1016/j.media.2019.101621 31816592 PMC7360136

[32] Lang Y, Wang L, Yap PT, Lian C, Deng H, Thung KH, et al. Automatic Detection of Craniomaxillofacial Anatomical Landmarks on CBCT Images Using 3D Mask R-CNN. In: Graph Learning in Medical Imaging. Cham: Springer International Publishing; 2019. p. 130–137.

[33] Lang Y, Lian C, Xiao D, Deng H, Yuan P, Gateno J, et al. Automatic Localization of Landmarks in Craniomaxillofacial CBCT Images Using a Local Attention-Based Graph Convolution Network. In: Medical Image Computing and Computer Assisted Intervention—MICCAI 2020. Cham: Springer International Publishing; 2020. p. 817–826. 10.1007/978-3-030-59719-1_79 PMC8675277 34927175

[34] Liu Q, Deng H, Lian C, Chen X, Xiao D, Ma L, et al. SkullEngine: A Multi-stage CNN Framework for Collaborative CBCT Image Segmentation and Landmark Detection. In: Machine Learning in Medical Imaging. Cham: Springer International Publishing; 2021. p. 606–614. 10.1007/978-3-030-87589-3_62 PMC8712093 34964046

[35] Chen R, Ma Y, Chen N, Liu L, Cui Z, Lin Y, et al. Structure-Aware Long Short-Term Memory Network for 3D Cephalometric Landmark Detection. IEEE Transactions on Medical Imaging. 2022;41(7):1791–1801. doi: 10.1109/TMI.2022.3149281 35130151

[36] Jiang Y, Li Y, Wang X, Tao Y, Lin J, Lin H. CephalFormer: Incorporating Global Structure Constraint into Visual Features for General Cephalometric Landmark Detection. In: Wang L, Dou Q, Fletcher PT, Speidel S, Li S, editors. Medical Image Computing and Computer Assisted Intervention—MICCAI 2022. Cham: Springer Nature Switzerland; 2022. p. 227–237.

[37] Swennen GR, Schutyser FA, Hausamen JE. Three-dimensional cephalometry: a color atlas and manual. Springer Science & Business Media; 2005.

[38] Serafin M, Baldini B, Cabitza F, Carrafiello G, Baselli G, Del Fabbro M, et al. Accuracy of automated 3D cephalometric landmarks by deep learning algorithms: systematic review and meta-analysis. La radiologia medica. 2023;128(5):544–555. doi: 10.1007/s11547-023-01629-2 37093337 PMC10181977

[39] Lang Y, Lian C, Xiao D, Deng H, Thung KH, Yuan P, et al. Localization of Craniomaxillofacial Landmarks on CBCT Images Using 3D Mask R-CNN and Local Dependency Learning. IEEE Transactions on Medical Imaging. 2022;41(10):2856–2866. doi: 10.1109/TMI.2022.3174513 35544487 PMC9673501

[40] Chen R, Ma Y, Liu L, Chen N, Cui Z, Wei G, et al. Semi-supervised anatomical landmark detection via shape-regulated self-training. Neurocomputing. 2022;471:335–345. doi: 10.1016/j.neucom.2021.10.109

[41] Chen X, Lian C, Deng HH, Kuang T, Lin HY, Xiao D, et al. Fast and accurate craniomaxillofacial landmark detection via 3D faster R-CNN. IEEE transactions on medical imaging. 2021;40(12):3867–3878. doi: 10.1109/TMI.2021.3099509 34310293 PMC8686670

[42] de Oliveira AEF, Cevidanes LHS, Phillips C, Motta A, Burke B, Tyndall D. Observer reliability of three-dimensional cephalometric landmark identification on cone-beam computerized tomography. Oral Surgery, Oral Medicine, Oral Pathology, Oral Radiology, and Endodontology. 2009;107(2):256–265. doi: 10.1016/j.tripleo.2008.05.039 18718796 PMC2642991

[43] Recht MP, Dewey M, Dreyer K, Langlotz C, Niessen W, Prainsack B, et al. Integrating artificial intelligence into the clinical practice of radiology: challenges and recommendations. European radiology. 2020;30:3576–3584. doi: 10.1007/s00330-020-06672-5 32064565

[44] Vokinger KN, Feuerriegel S, Kesselheim AS. Mitigating bias in machine learning for medicine. Communications medicine. 2021;1(1):25. doi: 10.1038/s43856-021-00028-w 34522916 PMC7611652

[45] Willemink MJ, Koszek WA, Hardell C, Wu J, Fleischmann D, Harvey H, et al. Preparing medical imaging data for machine learning. Radiology. 2020;295(1):4–15. doi: 10.1148/radiol.2020192224 32068507 PMC7104701

[46] Newell A, Yang K, Deng J. Stacked hourglass networks for human pose estimation. In: European conference on computer vision. Springer; 2016. p. 483–499.

[47] Virtanen P, Gommers R, Oliphant TE, Haberland M, Reddy T, Cournapeau D, et al. SciPy 1.0: Fundamental Algorithms for Scientific Computing in Python. Nature Methods. 2020;17:261–272. doi: 10.1038/s41592-019-0686-2 32015543 PMC7056644

[48] Xia JJ, Gateno J, Teichgraeber JF, Christensen AM, Lasky RE, Lemoine JJ, et al. Accuracy of the computer-aided surgical simulation (CASS) system in the treatment of patients with complex craniomaxillofacial deformity: a pilot study. Journal of oral and maxillofacial surgery. 2007;65(2):248–254. doi: 10.1016/j.joms.2006.10.005 17236929

[49] Junaid N, Khan N, Ahmed N, Abbasi MS, Das G, Maqsood A, et al. Development, application, and performance of artificial intelligence in cephalometric landmark identification and diagnosis: a systematic review. In: Healthcare. vol. 10. MDPI; 2022. p. 2454. 10.3390/healthcare10122454 PMC9778374 36553978

[50] Albalawi F, Alamoud KA. Trends and application of artificial intelligence technology in orthodontic diagnosis and treatment planning—A review. Applied Sciences. 2022;12(22):11864. doi: 10.3390/app122211864

[51] Leck R, Paul N, Rolland S, Birnie D. The consequences of living with a severe malocclusion: A review of the literature. Journal of Orthodontics. 2022;49(2):228–239. doi: 10.1177/14653125211042891 34488471 PMC9160782

[52] Damstra J, Fourie Z, H Slater JJ, Ren Y. Reliability and the smallest detectable difference of measurements on 3-dimensional cone-beam computed tomography images. American journal of orthodontics and dentofacial orthopedics. 2011;140(3):e107–e114. doi: 10.1016/j.ajodo.2011.02.020 21889058

