# Supplementary material for: Deep learning for 3D cephalometric landmarking with heterogeneous multi-center CBCT dataset
Source: PLoS One. 2024 Jun 25;19(6):e0305947. doi: 10.1371/journal.pone.0305947 (PMC11198780; doi:10.1371/journal.pone.0305947)
Supplement: S1 Appendix — (PDF) [file pone.0305947.s001.pdf]

Deep learning for 3D cephalometric landmarking with heterogeneous multi-center CBCT dataset

Jaakko Sahlsten, Jorma Järnstedt, Joel Jaskari, Hanna Naukkarinen, Phattaranant Mahasantipiya, Arnon Charuakkra, Krista Vasankari, Ari Hietanen, Osku Sundqvist, Antti Lehtinen, Kimmo Kaski.

Supplementary methods and results

1 Supplementary methods

1.1 Performance with partial volumes

In clinical settings some of the head scans include only parts of the full skull e.g., only including the lower jaw area. The dataset was augmented five times by cropping the original volumes by 25%, 50% and 75% from the left, right, bottom, and top directions as well as one with all these directions. In this experiment the performance was evaluated only with the landmarks within the cropped volume as the proposed deep learning system does not classify whether the landmark is visible or not.

1.2 Development environment

The original DICOM images were prepossessed by resampling, normalizing and extracting into NumPy arrays [1]. All the models were developed using PyTorch framework [2] on a machine with four GeForce GTX 1080 Ti GPUs and an Intel i7-6850K CPU. Statistical tests were calculated and applied to figures with Statannotations (v0.6) repository [3].

2 Supplementary results

Additional cohort comparison of landmarking when grouping by the group type, dosage, and location on bony surface is reported on Fig A. In addition, the supplementary results include boxplot figures for landmarking and cephalometric characteristic measures, both including the filtered annotation errors over ≥ 10 mm from the qualitative analysis, are shown on Fig B and C, respectively.

The overall mean error 1.97 ± 1.34 mm and successful detection rate (SDR) was 62.3 % for the 4683 landmarks in the 103 scans. The overall performance in partial volumes of the five different directions, the mean error was 2.02 ± 1.36 mm, 2.05 ± 1.48 mm and 2.36 ± 2.41 mm and the SDR value was 60.1 %, 59.1 % and 53.9 % for the 25%, 50% and 75% partial volumes, respectively. Cohort specific results are shown on Table A.

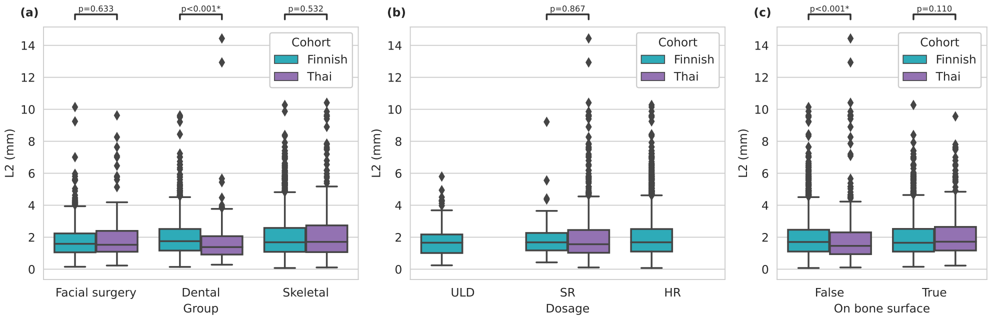

Fig A. Boxplot of grouped landmarking results. Grouped by (a) type, (b) dosage category and (c) landmark location on bony surface. Statistical significance was determined using the Mann-Whitney U test with Benjamini–Hochberg correction procedure. *⁾statistically significant difference.

Table A. Landmarking performance on partial volumes.

Cohort	Partial volume	Distance (mm)	SDR (%)	Landmarks
Finnish	0% × 1	1.96 ± 1.25	61.7	3717
	25% × 5	2.00 ± 1.25	59.6	17867
	50% × 5	2.06 ± 1.37	58.1	11684
	75% × 5	2.32 ± 2.11	53.2	2702
Thai	0% × 1	1.99 ± 1.55	64.3	966
	25% × 5	2.08 ± 1.73	62.1	4500
	50% × 5	2.03 ± 1.84	62.9	2889
	75% × 5	2.49 ± 3.23	56.3	748

Mean and standard deviation of landmarking distance and successful detection rate (SDR) for the partial volumes and the original dataset. Partial volumes are cut from the back, left, right, top and all directions.

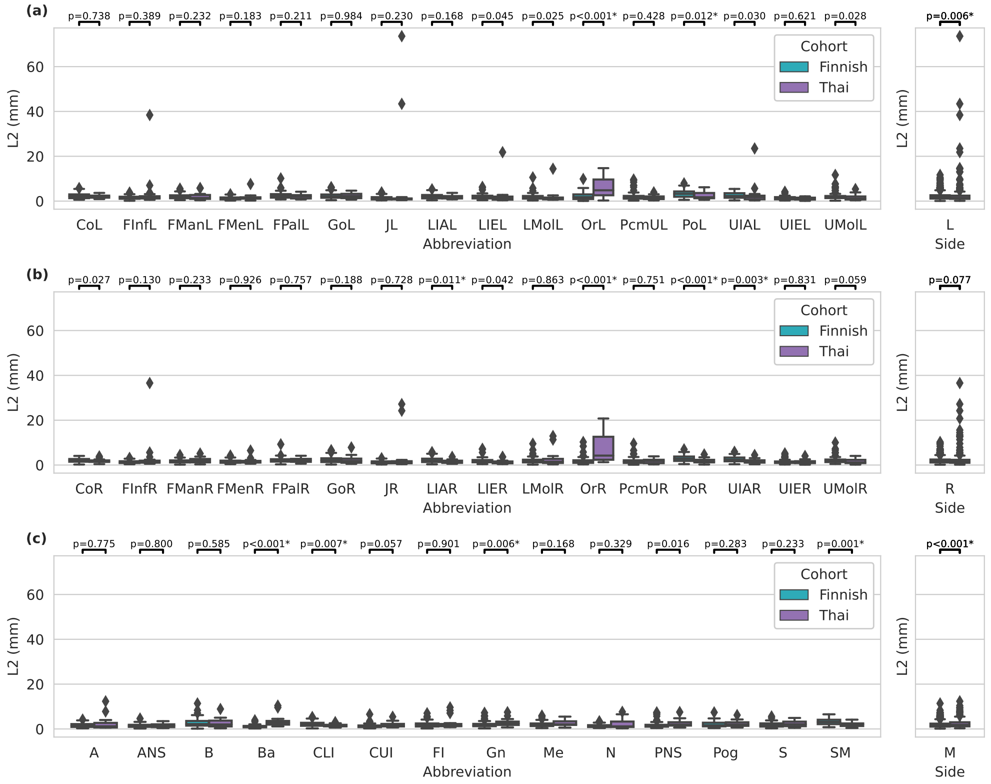

Fig B. Boxplot of landmarking results including annotation errors. Each row having (a) left, (b) right and (c) midline landmarks shown individually and as a group. Statistical significance was determined using the Mann-Whitney U test with Benjamini–Hochberg correction procedure. *⁾statistically significant difference.

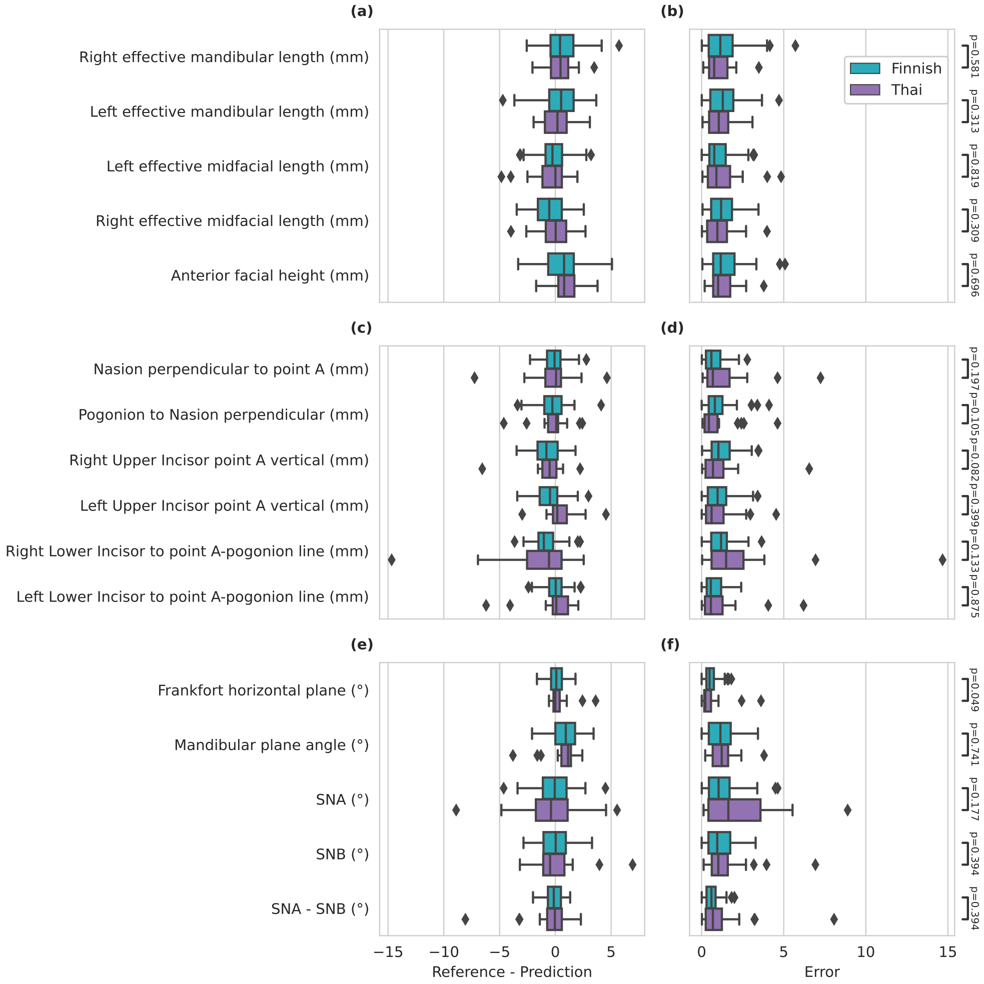

Fig C. Boxplot of performance on cephalometric characteristic measures including annotation errors with 3D point-to-point measures. (a) reference to prediction distance and (b) error, components of vector with (c) reference to prediction distance and (d) error, and plane-to-plane angle with (e) reference to prediction distance and (f) error. Cohort differences between each error measure are compared with the Mann-Whitney U test with Benjamini–Hochberg correction procedure. *⁾statistically significant difference.

References

1. Harris CR, Millman KJ, van der Walt SJ, Gommers R, Virtanen P, Cournapeau D, et al. Array programming with NumPy. Nature. 2020;585(7825):357–362. doi:10.1038/s41586-020-2649-2.

2. Paszke A, Gross S, Massa F, Lerer A, Bradbury J, Chanan G, et al. Pytorch: An imperative style, high-performance deep learning library. Advances in neural information processing systems. 2019;32.

3. Charlier F, Weber M, Izak D, Harkin E, Magnus M, Lalli J, et al.. Statannotations; 2022. Available from: https://doi.org/10.5281/zenodo.7213391.
